# Exploring Digital Biomarkers of Illness Activity in Mood Episodes: Hypotheses Generating and Model Development Study

**DOI:** 10.2196/45405

**Published:** 2023-05-04

**Authors:** Gerard Anmella, Filippo Corponi, Bryan M Li, Ariadna Mas, Miriam Sanabra, Isabella Pacchiarotti, Marc Valentí, Iria Grande, Antoni Benabarre, Anna Giménez-Palomo, Marina Garriga, Isabel Agasi, Anna Bastidas, Myriam Cavero, Tabatha Fernández-Plaza, Néstor Arbelo, Miquel Bioque, Clemente García-Rizo, Norma Verdolini, Santiago Madero, Andrea Murru, Silvia Amoretti, Anabel Martínez-Aran, Victoria Ruiz, Giovanna Fico, Michele De Prisco, Vincenzo Oliva, Aleix Solanes, Joaquim Radua, Ludovic Samalin, Allan H Young, Eduard Vieta, Antonio Vergari, Diego Hidalgo-Mazzei

**Affiliations:** 1 Department of Psychiatry and Psychology Institute of Neuroscience Hospital Clínic de Barcelona Barcelona, Catalonia Spain; 2 Bipolar and Depressive Disorders Unit Digital Innovation Group Institut d’Investigacions Biomèdiques August Pi i Sunyer (IDIBAPS) Barcelona, Catalonia Spain; 3 Biomedical Research Networking Centre Consortium on Mental Health (CIBERSAM) Instituto de Salud Carlos III Madrid Spain; 4 Department of Medicine School of Medicine and Health Sciences University of Barcelona (UB) Barcelona, Catalonia Spain; 5 Institute of Neurosciences (UBNeuro) University of Barcelona Barcelona, Catalonia Spain; 6 School of Informatics University of Edinburgh Edinburgh United Kingdom; 7 Barcelona Clinic Schizophrenia Unit Institut d’Investigacions Biomèdiques August Pi i Sunyer (IDIBAPS) Barcelona, Catalonia Spain; 8 Imaging of Mood- and Anxiety-Related Disorders (IMARD) Group Institut d’Investigacions Biomèdiques August Pi i Sunyer (IDIBAPS) Barcelona, Catalonia Spain; 9 Early Psychosis: Interventions & Clinical-detection (EPIC) Lab, Department of Psychosis Studies Institute of Psychiatry Psychology and Neuroscience King's College London London United Kingdom; 10 Center for Psychiatry Research Department of Clinical Neuroscience Karolinska Institutet Stockholm Sweden; 11 Department of Psychiatry, Centre Hospitalier Universitaire (CHU) Clermont-Ferrand University of Clermont Auvergne, Centre National de la Recherche Scientifique (CNRS), Clermont Auvergne INP Institut Pascal (UMR 6602) Clermont-Ferrand France; 12 Association Française de Psychiatrie Biologique et Neuropsychopharmacologie (AFPBN) Paris France; 13 Centre for Affective Disorders Institute of Psychiatry, Psychology & Neuroscience King's College London London United Kingdom

**Keywords:** depression, mania, bipolar disorder, major depressive disorder, machine learning, deep learning, physiological data, digital biomarker, wearable, Empatica E4

## Abstract

**Background:**

Depressive and manic episodes within bipolar disorder (BD) and major depressive disorder (MDD) involve altered mood, sleep, and activity, alongside physiological alterations wearables can capture.

**Objective:**

Firstly, we explored whether physiological wearable data could predict (aim 1) the severity of an acute affective episode at the intra-individual level and (aim 2) the polarity of an acute affective episode and euthymia among different individuals. Secondarily, we explored which physiological data were related to prior predictions, generalization across patients, and associations between affective symptoms and physiological data.

**Methods:**

We conducted a prospective exploratory observational study including patients with BD and MDD on acute affective episodes (manic, depressed, and mixed) whose physiological data were recorded using a research-grade wearable (Empatica E4) across 3 consecutive time points (acute, response, and remission of episode). Euthymic patients and healthy controls were recorded during a single session (approximately 48 h). Manic and depressive symptoms were assessed using standardized psychometric scales. Physiological wearable data included the following channels: acceleration (ACC), skin temperature, blood volume pulse, heart rate (HR), and electrodermal activity (EDA). Invalid physiological data were removed using a rule-based filter, and channels were time aligned at 1-second time units and segmented at window lengths of 32 seconds, as best-performing parameters. We developed deep learning predictive models, assessed the channels’ individual contribution using permutation feature importance analysis, and computed physiological data to psychometric scales’ items normalized mutual information (NMI). We present a novel, fully automated method for the preprocessing and analysis of physiological data from a research-grade wearable device, including a viable supervised learning pipeline for time-series analyses.

**Results:**

Overall, 35 sessions (1512 hours) from 12 patients (manic, depressed, mixed, and euthymic) and 7 healthy controls (mean age 39.7, SD 12.6 years; 6/19, 32% female) were analyzed. The severity of mood episodes was predicted with moderate (62%-85%) accuracies (aim 1), and their polarity with moderate (70%) accuracy (aim 2). The most relevant features for the former tasks were ACC, EDA, and HR. There was a fair agreement in feature importance across classification tasks (Kendall W=0.383). Generalization of the former models on unseen patients was of overall low accuracy, except for the intra-individual models. ACC was associated with “increased motor activity” (NMI>0.55), “insomnia” (NMI=0.6), and “motor inhibition” (NMI=0.75). EDA was associated with “aggressive behavior” (NMI=1.0) and “psychic anxiety” (NMI=0.52).

**Conclusions:**

Physiological data from wearables show potential to identify mood episodes and specific symptoms of mania and depression quantitatively, both in BD and MDD. Motor activity and stress-related physiological data (EDA and HR) stand out as potential digital biomarkers for predicting mania and depression, respectively. These findings represent a promising pathway toward personalized psychiatry, in which physiological wearable data could allow the early identification and intervention of mood episodes.

## Introduction

Mood disorders, including bipolar disorder (BD) and major depressive disorder (MDD), are ranked among the top 25 leading causes of disease burden worldwide [1] and are associated with recurrent depressive and manic episodes. Manic episodes are characterized by increased activity and self-esteem, reduced need for sleep, and expansive mood and behavior, whereas during depressive episodes, patients experience decreased energy and activity, sadness, low self-esteem, and social withdrawal [2-4]. These changes in mood, sleep, and activity during mood episodes translate to changes in physiological data that novel research-grade wearables can capture with high precision in real time [5,6]. Linking these digital signals with illness activity could potentially identify digital biomarkers [7].

Biomarkers are characteristics that are measured as an indicator of pathogenic processes (disease-associated biomarkers) or responses to an exposure or intervention (drug-related biomarkers) [8]. These can include molecular, histological, radiographic, or physiological characteristics. Digital biomarkers are objective, quantifiable, and physiological, and behavioral measures are collected using digital devices that are portable, wearable, implantable, or digestible [9]. Traditional biomarkers can be invasive and expensive to measure and are difficult to collect over time, thus giving an incomplete view of the complexity and dynamism of the disease. Alternatively, digital biomarkers are usually noninvasive, modular, and cheaper to measure, and they provide access to continuous and longitudinal measurements, both qualitative and quantitative. Moreover, they offer novel ways of measuring health status by providing perspectives into diseases that were unavailable before, which can supplement and enhance conclusions from traditional biomarkers [10]. Digital biomarkers have the potential to redefine diagnosis, improve the accuracy of diagnostic methods, enhance monitoring, and personalize interventions [11], leading to precision medicine, especially in psychiatric diseases [12].

In the last decade, there has been an exponential growth in the number of digital biomarker studies in the health domain, especially in cardiovascular and respiratory diseases [9]. Wearables are the most common type of digital devices used in digital biomarker studies, especially those incorporating accelerometer sensors that measure physical activity [13]. Wearable devices include wristbands, smartwatches, smart shirts, smart rings, smart electrodes, smart headsets, smart glasses, and so on. Wrist-worn devices are the most common type of wearable device in mental health studies and have shown to be effective in diagnosing anxiety and depression. However, none of the studies used it for treatment. The most commonly used category of data for model development was physical activity data, followed by sleep and heart rate (HR) data [14]. There are several areas in health care in which wearable devices have shown potential, including monitoring, diagnosis, treatment, and rehabilitation of diseases. Even though wearables have shown accurate activity-tracking measurements and are acceptable for users [15], including feasibility studies in people with mental health problems [16], their implementation in usual clinical practice is still challenging [17].

Wearables collecting actigraphy, the noninvasive method of monitoring human rest and activity [18], can capture altered sleep rhythms in remitted BD [19] and also depressive symptoms [20]. In addition, actigraphy data from wearables have shown to accurately predict mood disorder diagnoses and symptom change [21]. Moreover, wearables collecting blood pulse have shown differences in HR variability (HRV) between BD and healthy controls (HCs) [22], as well as between affective states in BD [23]. In addition, people with bipolar and unipolar depression and suicidal behavior have long shown autonomic alterations that can be captured as hyporeactive electrodermal activity (EDA) [24,25], and in recent years, research-grade wearables have incorporated sensors allowing continuous EDA collection [26]. With these upgrades, in the latest years, it is now feasible to monitor mood changes in patients with MDD [27] and also predict the presence and severity of depressive states in BD and MDD with promising accuracy using wearable physiological data [28]. Despite these promising results, the specific roles of these digital signals and their longitudinal potential to measure illness activity and treatment response in mood disorders are still unknown.

The conjuncture of advances in machine learning [29] and the improved precision of wearable devices [30] may help identify physiological patterns of illness activity in mood disorders. Firstly, considering this promising background, we explored whether physiological wearable data could predict the severity of an acute affective episode at the intra-individual level (aim 1) and the polarity of an acute affective episode and euthymia among different individuals (aim 2). Secondarily, we explored which physiological data were related to prior predictions, generalization across patients, and associations between affective symptoms and physiological data.

## Methods

### Study Design

**Figure 1:**
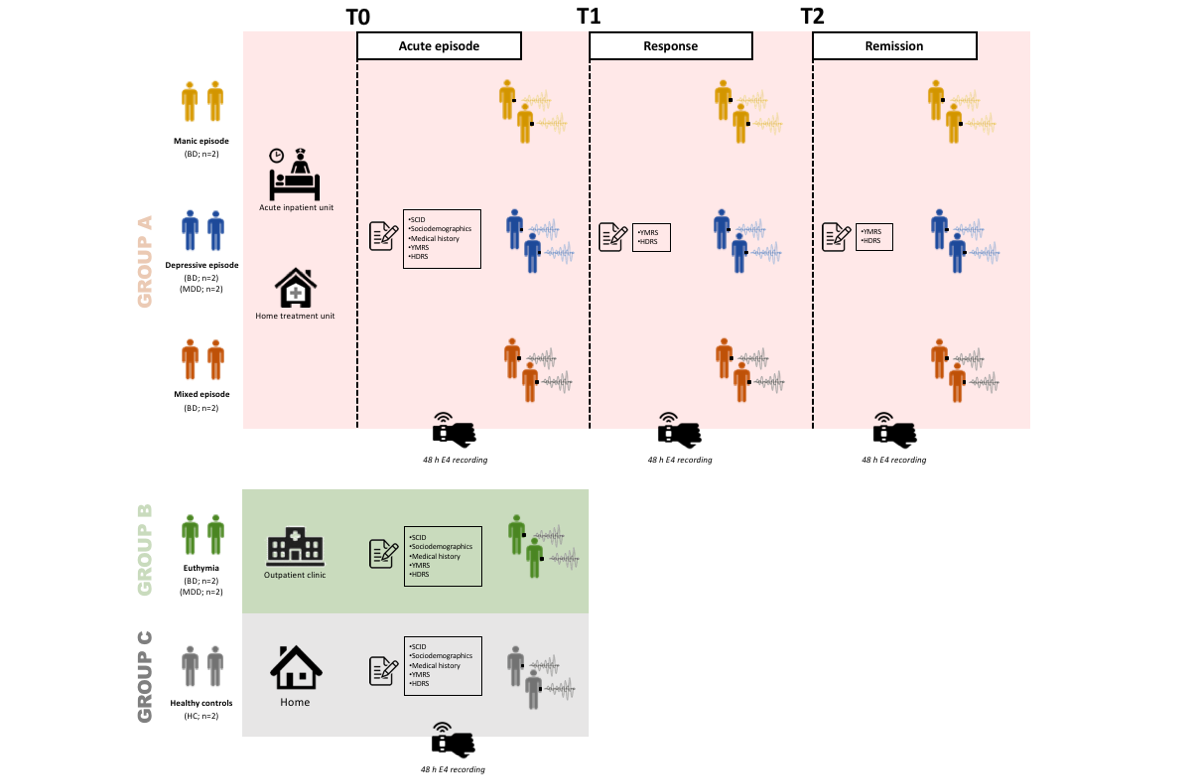
Study design and recordings. BD: bipolar disorder; HC: healthy controls; HDRS: Hamilton Depression Rating Scale; MDD: major depressive disorder; SCID: Structured Clinical Interview for Diagnostic and Statistical Manual of Mental Disorders; T0: current acute Diagnostic and Statistical Manual of Mental Disorders–5 affective episodes; T1: symptoms’ response; T2: symptomatic remission; YMRS: Young Mania Rating Scale.

A prospective exploratory observational study with 3 independent groups (Figure 1): group A, patients on acute affective episodes, manic episodes in BD (n=2), major depressive episodes in BD (n=2) and MDD (n=2), and mixed features manic episodes in BD (n=2); group B, euthymic patients with BD (n=2) and MDD (n=2); and group C, HC (n=7). Potential participants were identified at the outpatient and the acute inpatient or hospitalization at home units by their clinicians (ie, psychiatrists). Physiological data were recorded across 3 consecutive time points for group A: T0-acute (T0): current acute affective episodes according to the Diagnostic and Statistical Manual of Mental Disorders–5 (DSM-5); T1-response (T1): symptom response, as more than 30% improvement in the Young Mania Rating Scale (YMRS) score or the 17-item Hamilton Depression Rating Scale (HDRS) score; and T2-remission (T2): symptomatic remission, with YMRS and HDRS score ≤7 [31]). Euthymic patients (group B) and HCs (group C) were recorded during a single session.

The inclusion criteria were as follows: (1) aged above 18 years; (2) having a diagnosis according to the DSM-5 [32] criteria confirmed with the Structured Clinical Interview for DSM-5 Disorders [33]; and (3) willingness and ability to give consent (reconfirmed upon clinical remission). In addition, euthymic patients (group B) should also (4) score ≤7 on the YMRS and HDRS for at least 8 weeks [31]. HC (group C) should present no current or previous psychiatric disorder according to the DSM-5 criteria and confirmed using the Structured Clinical Interview for DSM-5 Disorders, excluding nicotine substance use disorder. Exclusion criteria for all groups were as follows: (1) concomitant severe cardiovascular or neurological medical conditions with a potential autonomic dysfunction, ongoing cardiovascular arrhythmia, or pacemaker; (2) comorbid current substance use disorder according to the DSM-5 criteria, excluding nicotine substance use disorder; (3) comorbid current psychiatric disorder with great interference of symptoms (eg, obsessive compulsive disorder with ritualized behaviors); (4) current pharmacological treatment with β-blockers or other pharmacological treatments affecting the autonomic nervous system; and (5) ongoing pregnancy.

### Assessments

The following sociodemographic variables were collected: age, sex, DSM-5 psychiatric diagnoses [32], medical and psychiatric comorbidities, years of illness duration, first-degree relative with mental illness, and drug misuse habits. Psychopathological assessments were conducted using the YMRS [34,35] for manic symptoms and the 17-item HDRS [36,37] for depressive symptoms. Clinical assessments were performed during a single session for euthymic patients (group B) and HCs (group C) and at 3 consecutive time points (T0-acute, T1-response, and T2-remission) for patients on acute affective episodes (group A), as described in Figure 1.

### Research-Grade Wearable Device for Recording

When choosing a wearable device for a research project, there are several factors that should be considered, including (1) the signals of interest to be captured (eg, stress-related and actigraphy); (2) the users who will be studied (eg, inpatients, outpatients, and HCs); (3) the pragmatic needs of the study (eg, budget, battery life, placement of the devices, and confidentiality of participants); (4) establishing assessment procedures (eg, stress elicitation task, resting, and sleep); and (5) performing qualitative and quantitative analyses on resulting data (eg, visually inspecting the data registered, quantifying data loss, assessing the quality of data, and comparing the data of different wearable devices) [38]. Considering the previous points, the E4 wristband from Empatica [39] was the preferred wearable device for the purpose of our study for several reasons. First, the E4 has shown accuracy in measuring HR, HRV [40], and EDA compared with laboratory conditions [41], as well as for sleep staging [42]. As previously mentioned, these physiological parameters have been shown to be altered in mood disorders and mood episodes [19-23,25-28]. Second, the E4 has been validated in scientific research for detecting emotional arousal, stress [43,44], and mental effort [45] using the aforementioned physiological signals. Furthermore, the E4 has proven to be useful in predicting depressive symptoms in MDD with low relative errors [46,47], predicting self-reported depressive states [48], and identifying and quantifying the severity of anxiety states [49]. In patients with BD, the E4 has shown to be useful in distinguishing manic from euthymic mood states [50,51]. Third, the inpatients included in the study were in a highly restricted setting, which would not allow the use of user-dependent wearables or devices providing external communication (eg, an internet connection). This requirement was fulfilled by the E4 device. Finally, the data recorded by the E4 are of high precision and quality [40,41], with minimal data loss when performing the analyses (see the *Results* section).

### Recording Procedure of Physiological Data

For each recording, patients and HCs were provided with an E4 wristband [39] (Multimedia Appendix 1) for approximately 48 hours (limited by battery life). The research team collected the wearables after each session. Individuals’ behavior was not externally influenced in any manner, further to the requirement of wearing the wristband. Patients with acute affective episodes (group A), during their psychiatric admission in the inpatient unit, were not allowed to leave the hospital at any point until discharge, as it is the standard practice with inpatients. T0-acute, T1-response, and T2-remission recordings were usually carried out in this setting. This was not the case with patients at the hospitalization at home or outpatient units (a minority of all cases), in which patients were not subject to mobility restrictions. In all cases, both for patients and HCs, participants were asked to wear the wristband during their daily life, with little to no interference in their behavior. They were also asked to put the wristband themselves at the beginning of the recording while researchers checked for adequate contact between the sensors and the skin wrist. Participants received instructions to remove the device when taking a shower to preserve the integrity of the device.

The E4 wristband has sensors that collect physiological data at different sampling rates. The physiological data signals from each recording session were collected from the following channels and sampling rates as raw data: 3D acceleration (ACC) in space over time on an x-, y-, and z-axis (ACC, 32 Hz); EDA (4 Hz); skin temperature (TEMP, 4 Hz); and blood volume pulse (BVP, 64 Hz); or in a processed format: interbeat intervals (IBIs, the time between 2 consecutive heart ventricular contractions) and HR (1 Hz). The BVP signal is obtained using a photoplethysmography sensor that measures volume changes in the blood. Empatica uses 2 algorithms on the BVP signal to construct an IBI with which HR (and HRV) can be calculated. The 2 algorithms are optimized to detect heartbeats and discard beats that contain artifacts [39,40].

### Preprocessing of Physiological Data

Owing to the naturalistic setting of the recording sessions, the data obtained from the E4 wristband are inherently noisy. For instance, some patients show low levels of compliance during an affective episode (eg, mania), which can lead to poor skin contact from the device, hence inaccurate readings for certain channels, or complete removal of the wearable device, resulting in unusable data. To that end, we removed invalid physiological data enforcing the rules-based filter by Kleckner et al [52] and an additional rule to remove HR values that exceed the physiologically plausible range (25-250 bpm) to quality control the raw data and remove physiologically impossible recordings (Table 1). Quality controlling physiological data from wearable devices is common practice, as this type of data is particularly noisy, and failing to quality control the data favors spurious correlations, and previous works have advised against imputing data in this scenario [53].

We did not use IBI data because of the disproportionately high number of missing values (approximately 70%) relative to data from different channels [54], especially because it is only a derivation of BVP. Therefore, we did not calculate HRV features. In sum, a total of 7 channels from the E4 device (ACC_X, ACC_Y, ACC_Z, BVP, EDA, HR, and TEMP) were used as physiological data to build the prediction models. Different time units (µ) and window lengths (w) were explored during tuning, and the best combination was selected. Because the sampling rate varied across different channels, the recordings were time aligned. If a channel’s sampling rate was higher than 1 Hz, that channel was downsampled by taking the average value across samples within µ. We compared different time units (µ=1, 2, 4, 32, and 64 Hz), and we used 1 Hz because it showed the best performance; therefore, a time unit µ=1 second was set across all channels. Upon time alignment, each recording was then segmented into a predefined number of segments using a tunable window length (w), taking values in real-time seconds (s) (only powers of 2, specifically from 2^0^ [1 s] to 2^11^ [2048 s], were explored for computational convenience). Of note, by tuning the hyperparameter w, an interesting pattern appeared across tasks, whereby a value of 2^5^ (ie, 32 s) emerged as an optimal point, whereas smaller or higher values were associated with a deterioration in validation performance (U-shaped performance); therefore, µ=1 Hz and w=2^5^ (32) seconds were used for analyses as the best-performing algorithm (Multimedia Appendix 2).

To obtain an equal number of segments from each class for model evaluation, we randomly selected 20 segments from each session and stored them as a held-out test set, which was never observed by the model during either training or validation. We then randomly assigned the remaining segments to the train and validation sets with ratios of 80% and 20%, respectively. Each segment was normalized (scaled to [0, 1]) using the per-channel global (across all segments) minimum and maximum values derived from the train set.

**Table 1:** Rules-based filter for invalid physiological data.

Rules	Filter for invalid data	Range
1	To prevent “floor” artifacts (eg, electrode loses contact with skin) and “ceiling” artifacts (circuit is overloaded)—EDA^a^ not in a valid range	0.05 to 60 µS^b^
2	EDA changes too quickly—EDA slope not in a valid range	−10 to +10 µS/second
3	Skin temperature suggests the EDA sensor is not being worn—skin temperature not in a valid range	30 to 40 °C
4^c^	HR^d^ not in a valid range	25 to 250 bpm^e^
5	Transitional data surrounding segments identified as invalid via the preceding rules—account for transition effects	Within 5 seconds

^a^EDA: electrodermal activity.

^b^µS: microsiemens.

^c^Addition to the algorithm used by Kleckner et al [52].

^d^HR: heart rate.

^e^bpm: beats per minute.

### Data Analyses

#### Tasks

The recording segments produced with the preprocessing steps described earlier were used in supervised learning experiments as input to the supervised models. For aim 1, models were trained on 3-class classification tasks (T0-acute, T1-response, and T2-remission) for each individual on an acute affective episode (manic BD, depressed BD, depressed MDD, and mixed BD). For aim 2, one model was trained on a 7-class classification task (manic BD, depressed BD, mixed BD, depressed MDD, euthymic BD, euthymic MDD, and HCs).

Segments from each class under a given task were extracted in the same number to obtain perfectly balanced classes. As sets were designed to be perfectly balanced, we adopted accuracy as our primary metric but also reported the *F*_1_-score, precision, and recall and computed the area under the receiver operating characteristic (AUROC) curves. It should be noted that ours is a multiclass setting, but as we had perfectly balanced sets, micro-, macro-, and weighted averages coincided. For the AUROC curves, the one-vs-rest multiclass strategy was adopted, also known as one-vs-all, which amounts to computing a receiver operating characteristic (ROC) curve for each class, so that at a given step, a given class is regarded as positive and the remaining classes are lumped together as a single negative class.

As part of our exploratory data analysis, to quantify the association between physiological data and affective symptoms measured by the YMRS and HDRS scale items, their normalized mutual information (NMI) was computed.

For each task, with the exception of the one about distinguishing members of a group of only HCs, as we were interested in testing the degree to which a model can generalize to different individuals, unseen during training, and sharing the same psychiatric label (diagnosis and psychopathological status), we prepared a test set of segments from recordings collected from an independent group of individuals. Therefore, the model was tested on this extra, independent holdout set to obtain an estimate of the out-of-sample generalization performance.

#### Model

We elected a Bidirectional Long Short-Term Memory (BiLSTM) model [55] as our model architecture. BiLSTM is a type of recurrent neural network (RNN), a class of deep learning model specifically designed to handle sequence data such as time series. RNNs process streams of data one time step at a time, and they store information regarding previous time steps in a hidden unit, such that the model output at each time step is informed by the current time step as well as by previous ones. Long short-term memory (LSTM) units represent an improvement over vanilla RNNs, as they address gradient instability by modeling the hidden state with cells that decide what to keep in memory and what to discard. This feature makes LSTM more efficient in capturing long-range dependencies. In contrast to a simple LSTM, BiLSTM reads the input sequence in 2 directions, from start to end and from end to start, thereby allowing for a richer representation. Although other deep learning architectures suitable for time series have been developed (more recently, the transformer [56]), as the aim of this work was exploratory rather than benchmarking different models, we contented ourselves with a single popular architectural choice for time series. By the same token, we used a simple shallow BiLSTM with 128 hidden units and tanh activation, followed by a single dense layer with softmax activation, to output the possible classes. The BiLSTM model was trained using the Adam optimizer [57] for 120 epochs with a learning rate of 0.001 and a batch size of 32 to minimize the cross-entropy between the ground-truth distribution over classes and the probability distribution of belonging to such classes outputted by the last network layer. To reduce overfitting, dropout [58] and early stopping were used. The choice of hyperparameters was based on a random search that yielded the best performance in the validation set.

#### Permutation Feature Importance

To assess the channels’ individual impact on the test set performance in the aforementioned tasks, we adopted a perturbation-based approach. For each channel at a time, we randomly permuted its values in the test set segments and computed the difference in performance relative to the baseline model. We chose this approach because it has a straightforward interpretation and provides a highly compressed, global insight into the importance of the channels. Agreement on channels’ relevance across different tasks was measured using the Kendall W.

### Code and Data Availability

The codebase was written in Python (version 3.8; Python Software Foundation), where the deep learning models were implemented in TensorFlow and developed on a single NVIDIA RTX 2080Ti. The repository for this study can be found on the internet [59].

### Ethics Approval and Confidentiality

This study was conducted in accordance with the ethical principles of the Declaration of Helsinki and Good Clinical Practice and the Hospital Clinic Ethics and Research Board (HCB/2021/104). All participants provided written informed consent before their inclusion in the study. All data were collected anonymously and stored encrypted in servers complying with all General Data Protection Regulation and Health Insurance Portability and Accountability Act regulations.

## Results

### Overview

A total of 35 sessions from 12 patients (manic, depressed, mixed, and euthymic) and 7 HCs (mean age 39.7, SD 12.6 years; 6/19, 32% female) were analyzed, totaling 1512 hours recorded. The median percentage of data per recording session dropped from further analysis of quality control was 11.05 (range 2.50-34.21). A clinical demographic overview of the study sample is presented in Table 2.

**Table 2:** Clinical demographic overview of the study sample.

Diagnosis	Age (years)	Sex	HDRS^a^ score			YMRS^b^ score		
			T0^c^	T1^d^	T2^e^	T0	T1	T2
Manic BD^f^	40	Male	5	4	4	24	8	2
Manic BD^g^	21	Male	3	5	4	23	15	1
Depressed BD^h^	33	Male	23	6	4	0	0	0
Depressed BD^g,h^	36	Male	17	12	3	2	4	2
Mixed BD	30	Female	8	4	4	30	20	5
Mixed BD^g^	40	Male	11	2	1	29	10	3
Depressed MDD^i^	57	Male	33	13	7	7	2	0
Depressed MDD^g^	45	Male	27	11	7	4	1	1
Euthymic BD	54	Male	3	—^j^	—	0	—	—
Euthymic BD^g^	61	Male	1	—	—	3	—	—
Euthymic MDD	60	Female	4	—	—	0	—	—
Euthymic MDD^g^	60	Male	3	—	—	0	—	—
HC^k^	32	Female	0	—	—	0	—	—
HC^g^	34	Male	0	—	—	0	—	—
HC	28	Female	0	—	—	1	—	—
HC	29	Male	0	—	—	2	—	—
HC	31	Male	2	—	—	1	—	—
HC	32	Female	1	—	—	3	—	—
HC	31	Female	0	—	—	1	—	—

^a^HDRS: Hamilton Depression Rating Scale.

^b^YMRS: Young Mania Rating Scale.

^c^T0: current acute Diagnostic and Statistical Manual of Mental Disorders–5 affective episodes or only register for euthymic patients and healthy controls.

^d^T1: symptoms’ response.

^e^T2: symptomatic remission.

^f^BD: bipolar disorder.

^g^The recording segments extracted from the marked subjects were used to check the models’ ability to generalize to clinically similar subjects, unseen during training.

^h^All registers performed at the hospitalization at home or outpatient units.

^i^MDD: major depressive disorder.

^j^Euthymic patients and healthy controls were recorded during a single session (T0).

^k^HC: healthy control.

### Aim 1: Prediction of the Severity of an Acute Affective Episode at the Intra-individual Level

The 3-class classification tasks (T0-acute, T1-response, T2-remission; accuracy expected by chance: 1/3=33%) to predict the severity of an acute affective episode showed accuracies ranging from 62% (depressed BD) to 85% (depressed MDD). The generalization models on unseen patients showed accuracies ranging from 28% (depressed MDD) to 57% (manic BD; Table 3). The confusion matrix is shown in Multimedia Appendix 3. This means that the model showed moderate to high accuracies for classifying the severity of each acute affective episode, with the best prediction models classifying individuals with depressed MDD and manic BD. However, generalization of the models was of very low accuracy for depressed MDD and mixed BD (by chance; approximately 30%), of low accuracy (slightly above chance; >40%) for mixed BD, and of moderate accuracy (>55%) for manic BD.

**Figure 2:**
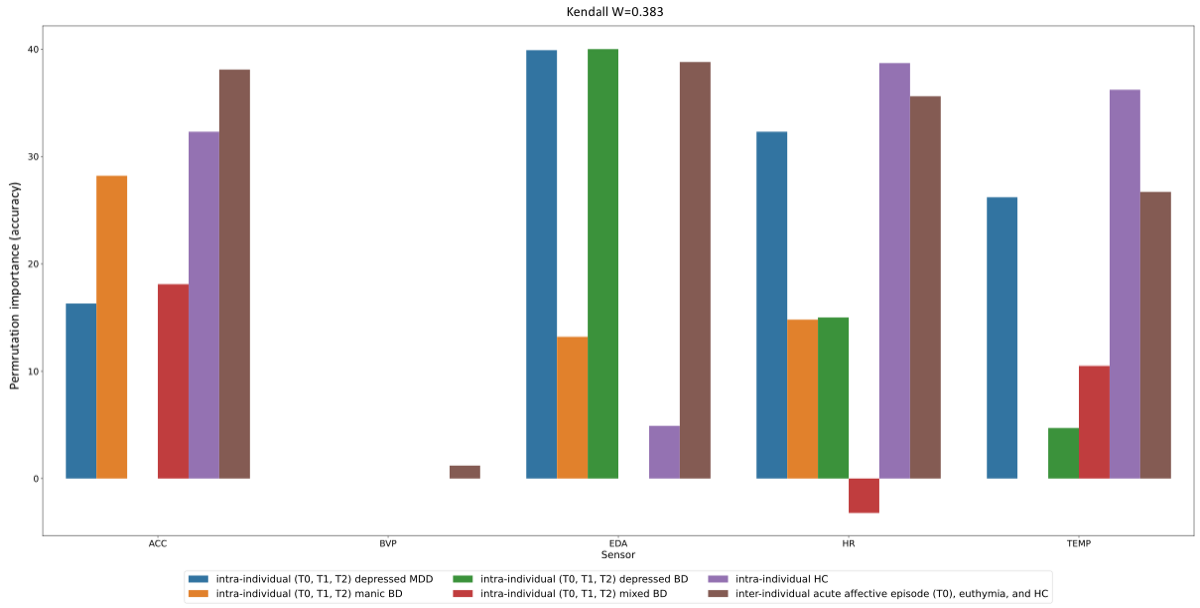
Permutation importance analysis. The height of the bars shows the change in accuracy at test time upon scrambling a channel through a random permutation of its values. A positive (negative) permutation importance value means that scrambling that channel results in a drop (increase) in accuracy relatively to the baseline where original (nonpermuted) values were used across all channels, that is, the channel’s permutation deteriorates (improves) the performance. A “0” permutation importance value indicates that a random permutation of the channel’s values does not affect accuracy in either direction. For instance, electrodermal activity (EDA) shows a positive change in accuracy of 40% for the intra-individual depressed BD severity prediction model; this means that removing this channel from the model would result in a decrease of prediction accuracy of 40%—from 62% to 22%—thus EDA is highly relevant for that model. Different colors correspond to the different tasks being investigated. ACC: acceleration; BD: bipolar disorder; BVP: blood volume pulse; HC: healthy controls; HR: heart rate; MDD: major depressive disorder; TEMP: temperature; T0: current acute Diagnostic and Statistical Manual of Mental Disorders–5 affective episodes; T1: symptoms’ response; T2: symptomatic remission.

The permutation importance analysis for the classification tasks for aims 1 and 2 is shown in Figure 2. Kendall W was 0.383, indicating fair agreement in feature importance across both intra- and inter-individual classification tasks. ACC was the most relevant channel for predicting mania, whereas EDA and HR, followed by TEMP, were the most relevant channels for predicting both BD and unipolar depression (aim 1). The BVP channel did not change performance for either better or worse (Figure 2).

**Table 3:** Prediction of the severity of an acute affective episode: model and generalization on unseen patients.

Individuals with affective episodes and performance metric		Model	Generalization
**Manic BD^a^**			
	Accuracy^b^ (%)	70	56.67
	*F*_1_-score	0.6978	0.5279
	Precision	0.6979	0.5381
	Recall	0.7000	0.5667
	AUROC^c^	0.6980	0.5432
**Depressed BD**			
	Accuracy^b^ (%)	61.67	41.67
	*F*_1_-score	0.6171	0.3968
	Precision	0.6273	0.4085
	Recall	0.6167	0.4167
	AUROC	0.6115	0.4067
**Mixed BD**			
	Accuracy^b^ (%)	63.33	30
	*F*_1_-score	0.6333	0.2576
	Precision	0.6333	0.3004
	Recall	0.6333	0.3068
	AUROC	0.6333	0.3012
**Depressed MDD^d^**			
	Accuracy^b^ (%)	85	28.33
	*F*_1_-score	0.8492	0.2451
	Precision	0.8774	0.2581
	Recall	0.8500	0.2833
	AUROC	0.8672	0.2856

^a^BD: bipolar disorder.

^b^Accuracy expected by chance for a 3-class classification task is 1/3=33%. Thus, accuracies above 33% suggest that the model can predict outcomes better than random guessing, and higher values for accuracy indicate better predictive capacity of the model. Note that the test set was designed to have the same number of samples in each class. This is reflected in the values of *F*_1_-score, precision, and recall being very close to each other and to that of accuracy.

^c^AUROC: area under the receiver operating characteristic.

^d^MDD: major depressive disorder.

### Aim 2: Prediction of the Polarity of an Acute Affective Episode and Euthymia Among Different Individuals

The 7-class classification task (accuracy expected by chance: 1/7=14%) to predict the polarity of affective episodes and euthymia showed an accuracy of 70%. The best classifications were depressed and euthymic MDD, followed by depressed BD, and the worst was manic BD, followed by HCs. The generalization model showed an accuracy of 15.7% (slightly above chance). The classification task for 7 HCs showed an accuracy of 50% (Table 4). The confusion matrix is shown in Multimedia Appendix 4. Thus, both models showed predictions above chance, but their generalization was poor. Moreover, the model including patients with acute affective episodes obtained higher accuracy (70%) than the model including 7 HCs (50%). This increased prediction capacity suggests that psychopathological symptoms during acute affective episodes may translate into physiological alterations that are not present in HCs.

The most relevant channels for predicting the polarity of affective episodes, euthymia, and HCs among different individuals (aim 2) were EDA, followed by ACC, HR, and TEMP (all channels showed >30% permutation importance). The BVP channel permutation importance was approximately 0%. These results were highly similar for the classification task of 7 HCs, but EDA showed only 4.9% permutation importance (Figure 2).

**Table 4:** Prediction of the polarity of an acute affective episode and euthymia among different individuals: model and generalization on unseen patients.

Individuals with affective episodes and performance metric		Model	Generalization
**6 patients (acute affective episodes and euthymia) and 1 HC^a^**			
	Accuracy^b^ (%)	70	15.7
	*F*_1_-score	0.6927	0.1516
	Precision	0.6889	0.1513
	Recall	0.6934	0.1517
	AUROC^c^	0.6900	0.1510
**7 HCs**			
	Accuracy^b^ (%)	50	—^d^
	*F*_1_-score	0.4923	—
	Precision	0.4911	—
	Recall	0.4988	—
	AUROC	0.4998	—

^a^HC: healthy control.

^b^Accuracy expected by chance for a 3-class classification task is 1/3=33%. Thus, accuracies above 33% suggest that the model can predict outcomes better than random guessing, and higher values for accuracy indicate better predictive capacity of the model. Note that the test set was designed to have the same number of samples in each class. This is reflected in the values of *F*_1_-score, precision, and recall being very close to each other and to that of accuracy.

^c^AUROC: area under the receiver operating characteristic.

^d^As we were interested in predicting affective psychopathology, we tested the degree to which a model can generalize to different individuals for each task except for the one about distinguishing members of a group of only HCs.

### Symptom Association With Physiological Data

**Figure 3:**
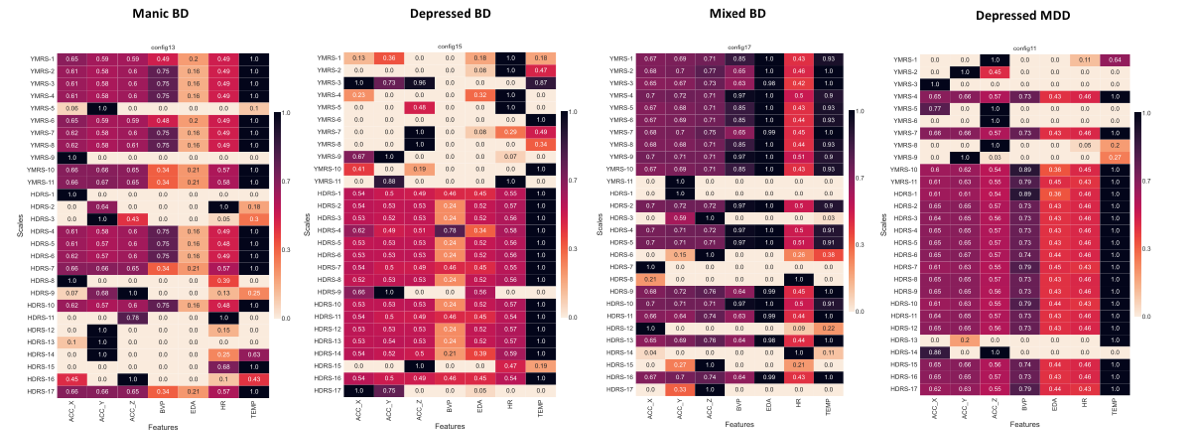
Tile plots for the normalized mutual information analysis between physiological data and psychometric scales’ items: intra-individual level. For each scales’ item the mutual information (MI) with respect to each of the channels was measured and scaled to 0 to 1 dividing by the maximum MI value for that item. Values of zero indicate no associations, values of 1 indicate the maximum recorded MI across all channels for an individual item. ACC_X: x-axis acceleration; ACC_Y: y-axis acceleration; ACC_Z: z-axis acceleration; BD: bipolar disorder; BVP: blood volume pulse; EDA: electrodermal activity; HDRS: Hamilton Depression Rating Scale; HR: heart rate; MDD: major depressive disorder; TEMP: temperature; YMRS: Young Mania Rating Scale.

**Figure 4:**
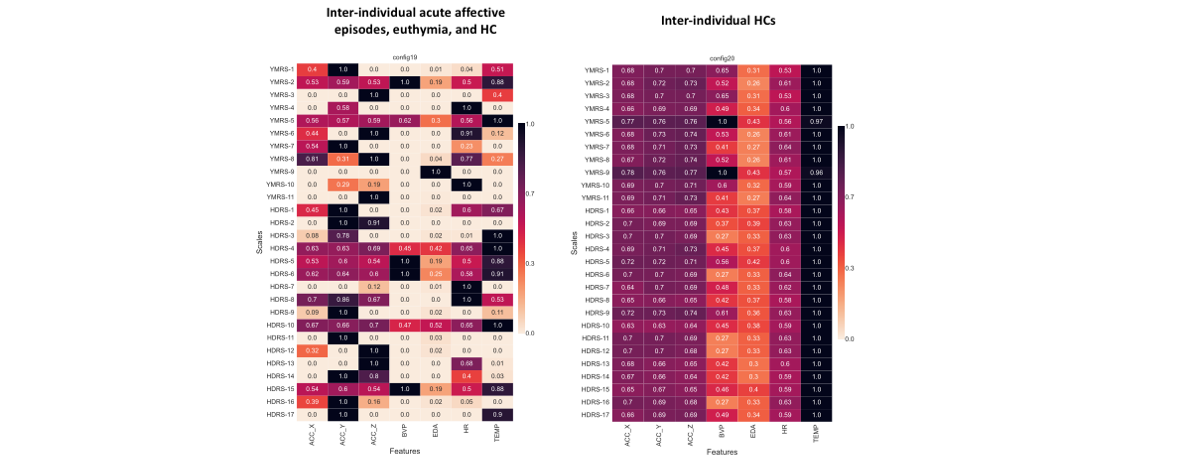
Tile plot for the normalized mutual information analysis between physiological data and psychometric scales’ items: between-individual level. For each scales’ item, the mutual information (MI) with respect to each of the channels was measured and scaled to 0 to 1 dividing by the maximum MI value for that item. Values of “0” indicate no associations; values of 1 indicate the maximum recorded MI across all channels for an individual item. ACC_X: x-axis acceleration; ACC_Y: y-axis acceleration; ACC_Z: z-axis acceleration; BVP: blood volume pulse; EDA: electrodermal activity; HC: healthy controls; HDRS: Hamilton Depression Rating Scale; HR: heart rate; TEMP: temperature; YMRS: Young Mania Rating Scale.

The tile plots for the NMI between physiological data and the YMRS and HDRS scale items for the former intra-individual (aim 1) and between-individuals (aim 2) classification tasks are shown in Figures 3 and 4, respectively. TEMP had the highest association with psychometric scales (NMI approximately 1.0), and BVP had the lowest consistency (NMI scores oscillating from 0 to 1).

#### Intra-individual NMI Analysis

Motor activity (ACC) channels were highly associated with manic symptoms (NMI>0.6), and stress-related channels (EDA and HR) with depressive symptoms (NMI from 0.4 to 1.0), as shown in Figure 3.

#### Between-Individuals NMI Analysis

“Increased motor activity” (YMRS item 2 [YMRS2]) was associated with ACC (NMI>0.55), “aggressive behavior” (YMRS9) with EDA (NMI=1.0), “insomnia” (HDRS4-6) with ACC (NMI∼0.6), “motor inhibition” (HDRS8) with ACC (NMI∼0.75), and “psychic anxiety” (HDRS10) with EDA (NMI=0.52), as shown in Figure 4.

## Discussion

### Principal Findings

Although other studies have used raw physiological data to predict mental health status, this is the first study to present a novel fully automated method for the analysis of raw physiological data from a research-grade wearable device, including a rules-based filter for invalid physiological data, whereas all other studies presented methods that required manual interventions at some point in the pipeline [46,47,51,60], thus hindering the replicability and scalability of results. Moreover, our preprocessing pipeline is strictly based on the best-performing algorithm for analysis (ie, not arbitrarily decided), whereas other studies decided arbitrary cutoff points for analyzing raw physiological data (eg, ACC data recorded at 32 Hz sampling rates analyzed arbitrarily in 1-min epochs [50]). Our method may allow other research teams to use a viable supervised learning pipeline for time-series analyses for a popular research-grade wristband [39]. In addition, our work integrates physiological digital data from all sensors captured by a research-grade wearable, and we assessed the relevance of each channel (ACC, TEMP, BVP, HR, and EDA) in the prediction models. In contrast, other studies have focused on specific digital signals, such as actigraphy [50], or used combinations of digital signals (such as actigraphy and EDA) and predesigned features (eg, amplitude of skin conductance response peaks) [51] but arbitrarily disregarded other digital signals, such as TEMP, or derived features, such as HRV. Furthermore, we aimed to distinguish the severity of mania and depression in a progressive and longitudinal manner according to the usual clinical resolution of mood episodes. We believe that the potential quantification of affective episodes is harder but a clinically more relevant task that may allow a more accurate and precise understanding of the disease rather than a mere dichotomous (acute vs remission) classification, as done in previous studies [50,51]. In addition, we included in the same work analyses at the intra-individual level and between different individuals, analyses targeting specific mood symptoms and generalization of the models on unseen patients. We believe that the use of different analysis methods allows us to examine the data from complementary perspectives to answer specific research questions. In addition, these different approaches may reveal random associations or artifacts that would stay hidden without replication. On the basis of these exploratory results, we propose hypotheses for future testing [61] in current and other similar projects.

**Figure 5:**
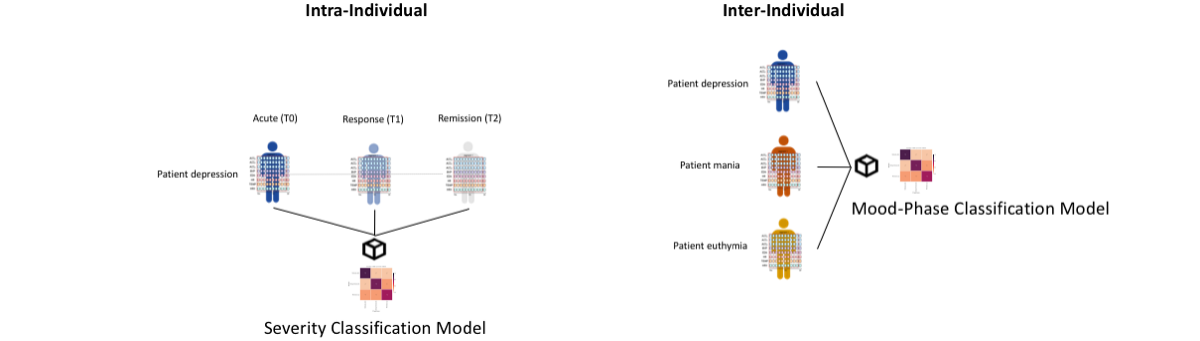
Severity versus Mood-Phase Classification Models: visual grounds for both intra- and inter-individual analyses. On the left, a severity classification model for a patient with depression (acute-response-remission phases). On the right, a mood-phase classification model (depression, mania, and euthymia). Note that on the left model, the same individual is compared at 3 different states (corresponding to a reduction in depressive psychopathology). Thus, individual-level characteristics (age, sex, and gait) should go through little to no variation across; should remain the same on the 3 longitudinal registers; and therefore, the shift in the covariate distribution should be relatively contained and not influence the classification of the model (capturing mood-relevant signals). In contrast, on the right, 3 different individuals at 3 different mood states are compared. In this case, the model would potentially distinguish between mood phases (mania vs depression), or cases from healthy controls, but may not be able to distinguish longitudinal changes in disease severity over the course of an index episode. In addition, in the latter model, subject-specific characteristics may be overlapped with mood-relevant signals, thus acting as confounders for the model. T0: current acute Diagnostic and Statistical Manual of Mental Disorders–5 affective episodes; T1: symptoms’ response; T2: symptomatic remission.

Note that both (1) intra- and (2) inter-individual analyses approach different research questions: the (1) intra-individual analytical approach looks at the course of an index episode within a single patient and examines whether different states (from the acute phase to response and remission) can be distinguished from each other; on the other hand, the (2) inter-individual analytical approach takes a cross-sectional view and studies the degree to which different mood disorder states (comprising the full spectrum from depression to mixed state, mania, and euthymia) can be separated. Both analyses try to identify digital biomarkers of illness activity using physiological data collected with a wristband. However, intra-individual analyses look for a fine-grained quantification of illness activity that may allow the identification of low-severity mood states (or prodromal phases) in comparison with moderate to severe ones. Conversely, inter-individual analyses could potentially distinguish between mood phases (mania vs depression) or cases from HCs but may not be suitable for assessing the severity of mood episodes, as represented in Figure 5. Studies in similar areas, such as brain computer interfaces for the rehabilitation of motor impairments [62] or seizure forecasting [63], emphasized the importance of the subject-wise approach (modeling each subject separately). In many instances, despite work on domain adaptation [64] to learn subject-invariant representations, a model has to be fine-tuned to the level of the single patient.

Studies comparing intra- and inter-individual models show that although intra-individual (cross-subject or patient-specific) models are trained on the data of a single subject, they perform better than intersubject (within-subject or generalized) models [65]. However, some studies have shown that hybrid models trained on multiple subjects and then fine-tuned on subject-specific data led to the best performance, without requiring as much data from a specific subject [66]. In intersubject studies, models generally see more data, as multiple subjects are included, but must contend with greater data variability, which introduces different challenges. In fact, there is both intra- and intersubject variability owing to time-variant factors related to the experimental setting and underlying psychological parameters. This impedes direct transferability or generalization among sessions and subjects [62]. To illustrate this, in a study aimed at evaluating a seizure detection model using physiological data and determining its application in a real-world setting, 2 procedures were applied: intra- and intersubject evaluation. Intrasubject evaluation focuses on the performance of the methodology when applied to data from a single patient, whereas intersubject evaluation assesses the performance of multiple patients with potentially different types of epilepsy and seizure manifestations [63].

Notably, the out-of-sample generalizations of both models differ vastly. Whereas the intra-individual model requires multiple seizures recorded per subject and will produce individualized models tailored to a single patient, the inter-individual model requires seizures recorded from multiple participants and will provide intersubject models to be used over wider populations. For this purpose, intersubject variability plays a key role: focal seizures have a multitude of possible clinical manifestations that can occur in sequence or in parallel and can be repeated or not occur at all, in a single seizure. For instance, preictal tachycardia appears to be a phenomenon that is not generalizable to patient cohorts. Furthermore, although there may oftentimes be little change in the semiology of seizures for a single patient, they can be very heterogeneous across populations. Intra-individual models optimized for each patient can robustly detect seizures in some patients with epilepsy, but they may fail, especially when the seizures have differing semiologies that are not represented in the training data for the model. Intersubject models perform worse than if trained in an individualized manner, at least in terms of either sensitivity or false-alarm rates [63]. This is equivalent to a study aimed at evaluating a model for mood episode detection and determining its application in a real-world setting. During acute affective episodes, a huge combination of symptoms can be present in 2 different patients [67,68], and recurrent longitudinal affective episodes in a single patient can present with a similar combination of symptoms, but this is not always the case [69-72]. At the intrasubject level, out-of-sample generalization would require multiple episodes of disease occurrence longitudinally in a single patient. In fact, similar studies with intra-individual models have achieved high detection accuracies with low sample sizes and better performance than intersubject classification [63,73]. In contrast, at the intersubject level, out-of-sample generalization does not require longitudinal episodes but only cross-sectional episodes in different patients. Therefore, both models serve different but complementary purposes to build a real-world model for the detection of prodromal affective symptoms. Future studies combining intra- and inter-individual analyses should determine which of these approaches may work best to identify affective episodes, giving guidance for the design of future studies in the field.

Clinically, the end goal is to have a model inferring mood states at the individual level, regardless of whether such a model is shared across subjects or if each subject has a tailored model. Although most digital biomarker research has focused on diagnosis classification, few studies have aimed to detect longitudinal symptom change. Developing methods to detect changes in mood symptoms has the potential to prompt just-in-time interventions to prevent full-blown affective relapses and clinical deterioration and evaluate the response to pharmacological treatments with objective measures [21].

In our sample, both intra- and inter-individual models for respectively assessing differences in severity of acute affective episodes over time (Table 3) and differences in the polarity of acute affective episodes, euthymia, and HCs (Table 4) showed accuracies considerably above chance. Although preliminary, these results indicate that there may be objective differences in digital signals (ie, digital biomarkers) according to the psychopathological severity of patients (intra-individual models) and that patients with BD or MDD may present particular patterns of digital signals for mood episodes of mania and depression (inter-individual models). However, with few patients and measurements per model, these digital biomarkers may be challenging to identify and even harder to generalize.

Motor activity (from ACC) was the most relevant digital signal for predicting the severity of mania and mixed mania (but not for unipolar or bipolar depression) and also for predicting the polarity of acute affective episodes between individuals (Figure 2). In line with our results, other research groups have found that wearable motor activity data can distinguish mania from remission in patients with BD at the intra-individual level [50]. Moreover, other studies have shown that motor activity data could identify mood episodes and euthymia among different individuals, including mania versus euthymia [51], depression versus HCs [60], and mania versus depression versus HCs [74]. In fact, “activation,” which comprises having objective (motor activity) and related subjective (energy) levels emerging from underlying physiological changes, has been widely recognized as a key feature from mania [75]. Previous literature proposes that mood and activation represent distinct dimensions of BD [76] with distinct intervention approaches [77]. In addition, dysregulation of patterns of activity has been observed in BD both in acute phases and euthymia and has been proposed as a potential biomarker for BD [78]. However, it should be noted that mania may be better characterized by differences in robustness, variability, predictability, or complexity of activation rather than mean levels of activity [75], so future analyses should explore which characteristics of motor activity are key for the former predictions.

In contrast, “stress-related” digital signals (EDA and HR) were the most relevant for predicting the severity of both unipolar and bipolar depression (but not mania or mixed mania) and were also prominent for predicting the polarity of acute affective episodes between individuals (Figure 2). In fact, when looking at psychic anxiety as a symptom (item 10 from HDRS), EDA and HR showed strong associations (Figure 4). Moreover, EDA showed relevance for predicting the polarity of affective episodes between individuals but did not differentiate between HCs (38% vs 4.9%), as shown in Figure 2. This suggests that EDA may be a specific marker for psychopathological alterations that are not present in HCs. Furthermore, skin TEMP (a proposed marker of stress) was also a relevant physiological signal for predicting the severity of unipolar and bipolar depression (Figure 2). These findings are in line with previous literature [26,79-82] and reinforce the hypothesis that stress plays a key role in people with depression. Whereas patients with manic episodes usually lack insight into their symptoms, patients with depression are usually aware of their altered state and bear much distress and anxiety [83], which may be translated into physiological alterations, as suggested in our findings.

Generalizations of the former models on unseen patients were of overall low accuracy, which may be due to high psychopathological and individual heterogeneity, as well as external factors. Although mood episodes share many psychopathological aspects, they can present with multiple combinations of symptoms [68,76,84]. Each digital signal may provide information on a specific symptom dimension (altered motor activity, sleep disturbances, and stress-related symptoms) rather than the entire affective episode (manic, depressive, or mixed). We hypothesized that training the models with a larger sample, including patients with different symptom combinations for each affective episode, will result in more precise generalizations. Thus, exploring how patients cluster according to physiological data might help toward a dimensional (rather than categorical) disease classification. Deep learning is a promising approach for clustering high-dimensional, unstructured data [85], and new methods have been proposed specifically for data from wearable devices (multivariate time series) [86,87]. Apart from polymorphic psychopathological presentations in mood episodes, there is high between-subject heterogeneity in physiological data. For instance, skin TEMP, HR, and EDA vary within a physiological range in the same individual according to external (ie, atmospheric humidity or ambient TEMP) or internal factors (ie, hydration, diet, caffeine intake, and drugs) [52], and there are also individual-level patterns (eg, specific gaits, circadian rhythms, basal skin TEMP, or HR). This calls for ad-hoc techniques to disentangle between-patient heterogeneity from mood-related signals [88] and consider the role of potential confounders in the models (eg, drugs, medical comorbidities, physical activity, atmospheric conditions, and diet). Notwithstanding, generalizations of the intra-individual models for manic BD and depressed BD were above chance, in contrast to the generalization of the inter-individual model (almost by chance). This may suggest that individual heterogeneity is partially controlled for when comparing the same individual at different time points. This way, physiological changes may be more related to psychopathology rather than simply to individual characteristics (eg, gait, sex, and age) However, intra-individual comparisons do not control for external factors (eg, humidity, atmospheric TEMP, exercise, or hydration), which should be considered and controlled for.

When exploring the association between affective symptoms and physiological data, skin TEMP showed the highest association with psychometric scales (NMI approximately 1.0; Figures 3 and 4). Skin TEMP has been proposed as an objective physiological marker of stress [89,90], and it has been shown that people with mood disorders present objective reductions in peripheral skin TEMP (due to vasoconstriction) after stress-oriented interventions [91]. Moreover, skin TEMP from wearable data has been used to study circadian rhythms in patients with mood disorders, showing alterations in their chronobiology [92]. Even so, thermoregulatory dysfunction has been proposed in a subgroup of patients with BD [93]. However, the skin TEMP continuously recorded with wearables has been relatively understudied in mood disorders, and further efforts should be made in this direction.

Regarding the most relevant inputs for the previous models, physiological data related to specific symptom dimensions (eg, ACC with motor activity and EDA and HR variation with stress response or anxiety) seemed to be more relevant signals for predicting mood episode severity and polarity rather than more raw data, such as BVP with nearly 0% permutation importance in all models (Figures 2-4), which do not seem to have a direct clinical translation to physiological alterations related to mental health symptoms. We hypothesized that complex features with potential clinical translation (ie, indicating stress response or autonomic dysfunction), such as HRV [22,23,94], which is calculated from BVP, and EDA reactivity, calculated from EDA [26], may be of greater value than second-to-second changes in motor activity (ACC), EDA, pulse (BVP), and TEMP. We hypothesized that adding derived features as input to the models will probably result in better predictions, as shown by other research groups when identifying mood states in BD using the same wristband device [51]. Therefore, we are currently exploring derived features from raw data (ie, statistical, time-domain, and frequency-domain features) [53], assessing EDA reactivity by extracting information on the tonic and phasic components of skin conductance using novel automated methods [18,53,95], and performing stress elicitation to assess potential alterations (hyporeactivity) in the phasic component of EDA during mood episodes [26]. Finally, considering the sleep and circadian rhythm disturbances in mood disorders in both euthymia [19,96] and acute phases [97-99], we are exploring automated methods to separate sleep from wake times [87,100,101]. Our goal is to evaluate sleep disturbances and differences in physiological signals during sleep and wake periods during mood episodes [77].

### Limitations

We acknowledge several limitations in this study. First, the limited sample size for model development does not allow us to make strong claims about generalization performance [102]. However, most recordings were longer than 40 hours and each patient on an acute mood episode was recorded longitudinally at 3 time points (acute, response, and remission). In fact, our data set in terms of recording hours is well above other data sets modeled with deep learning in health care settings: the deep convolutional approach proposed by Musallam et al [103] was applied to 60 hours of electroencephalogram recordings [104]. In addition, the wearable device used (E4), allows fine-grained collection of digital physiological data (from 1 Hz to 64 Hz) for precision longitudinal time-series analyses. Regarding sample size in terms of the number of subjects, previous endeavors used as few as 12 subjects [46]. Unfortunately, this type of data, that is, recorded with a research-grade wearable device on a population with a psychiatric condition (arguably interfering with compliance to instructions), is expensive and time-consuming to collect. Second, potential confounding variables such as sex, age, pharmacological treatments, exercise, or BMI were not controlled for, and some of the study sample was not matched by age and sex. This may have biased the results, as those variables have been found to affect motor activity data, especially in between-group comparisons [60]. The within-subject design allows partial mitigation of both the weakness of a small sample size and the influence of confounders, so the models can capture mood-related signals. Therefore, we performed intra-individual comparisons across consecutive time points. In fact, the generalization of intra-individual models obtained substantially better accuracies, showing glimpses of capturing the severity of manic and depressive psychopathology.

Future works will further explore the capabilities of advanced automated machine learning models for identifying affective illness activity and the role of confounders in this association. Of particular interest are the application of clustering algorithms [87], exploring derived features (HRV [94] and EDA reactivity [26]), the role of wake and sleep periods [77,105], and the potential of physiological data to predict treatment responses and detect prodromal signs of mood episodes [106]. Future projects will include (1) studying the role of psychotic symptoms in patients with affective disorders, as well as in patients with schizophrenia; (2) assessing the role of smartphone-based derived data, including ecologic momentary assessments and passive data [107-109], in patients with BD using the SIMPLe smartphone app [110,111]; and (3) investigating the potential of combining physiological wearable data with peripheral biomarkers [112,113] and speech features [114-118].

### Conclusions

Physiological wearable data may have the potential to identify and predict the severity of mania and depression in mood disorders as well as specific symptoms quantitatively. Motor activity appears to be the most relevant digital biomarker for predicting mania, whereas stress-related digital biomarkers (EDA and HR) appear to be the most relevant for predicting both bipolar and unipolar depression. In the context of biomarkers in mood disorders, these findings represent a promising pathway toward personalized psychiatry, in which clinical decisions and treatments could be supported by passive continuous and objective digital data.

## Appendix

Multimedia Appendix 1 Empatica E4.

Multimedia Appendix 2 Validation set performance (accuracy) as a function of time alignment (Hz) and window length (w).

Multimedia Appendix 3 Confusion matrix for the prediction of the severity of an acute affective episode: models and generalization. BD: bipolar disorder; MDD: major depressive disorder.

Multimedia Appendix 4 Confusion matrix for the prediction of the polarity of affective episodes, euthymia, and healthy controls: models and generalization. BD: bipolar disorder; HC: healthy controls; MDD: major depressive disorder; T0: current acute Diagnostic and Statistical Manual of Mental Disorders–5 affective episodes.

## Abbreviations

ACC: acceleration
AUROC: area under the receiver operating characteristic
BD: bipolar disorder
BiLSTM: Bidirectional Long Short-Term Memory
BVP: blood volume pulse
DSM-5: Diagnostic and Statistical Manual of Mental Disorders–5
EDA: electrodermal activity
HC: healthy control
HDRS: Hamilton Depression Rating Scale
HR: heart rate
HRV: heart rate variability
IBI: interbeat interval
LSTM: long short-term memory
MDD: major depressive disorder
NMI: normalized mutual information
RNN: recurrent neural network
ROC: receiver operating characteristic
T0: current acute Diagnostic and Statistical Manual of Mental Disorders–5 affective episodes
T1: symptoms' response
T2: symptomatic remission
TEMP: temperature
YMRS: Young Mania Rating Scale

## Data Availability

The data supporting the findings of this study are available upon request from the corresponding author.
