# Supplementary figures and images for: Exploring Digital Biomarkers of Illness Activity in Mood Episodes: Hypotheses Generating and Model Development Study
Source: JMIR Mhealth Uhealth. 2023 May 4;11:e45405. doi: 10.2196/45405 (PMC10196899; doi:10.2196/45405)

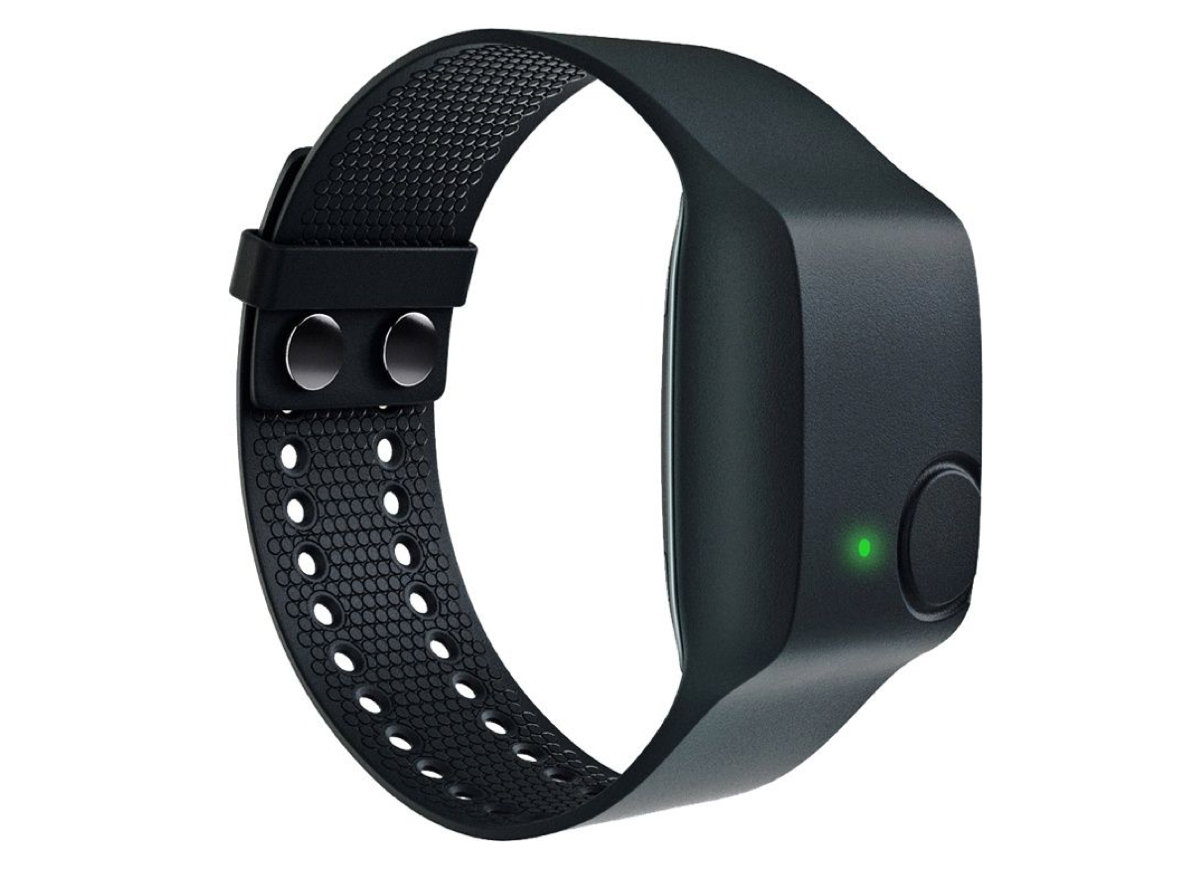

Supplement: Multimedia Appendix 1 [file mhealth_v11i1e45405_app1.png]

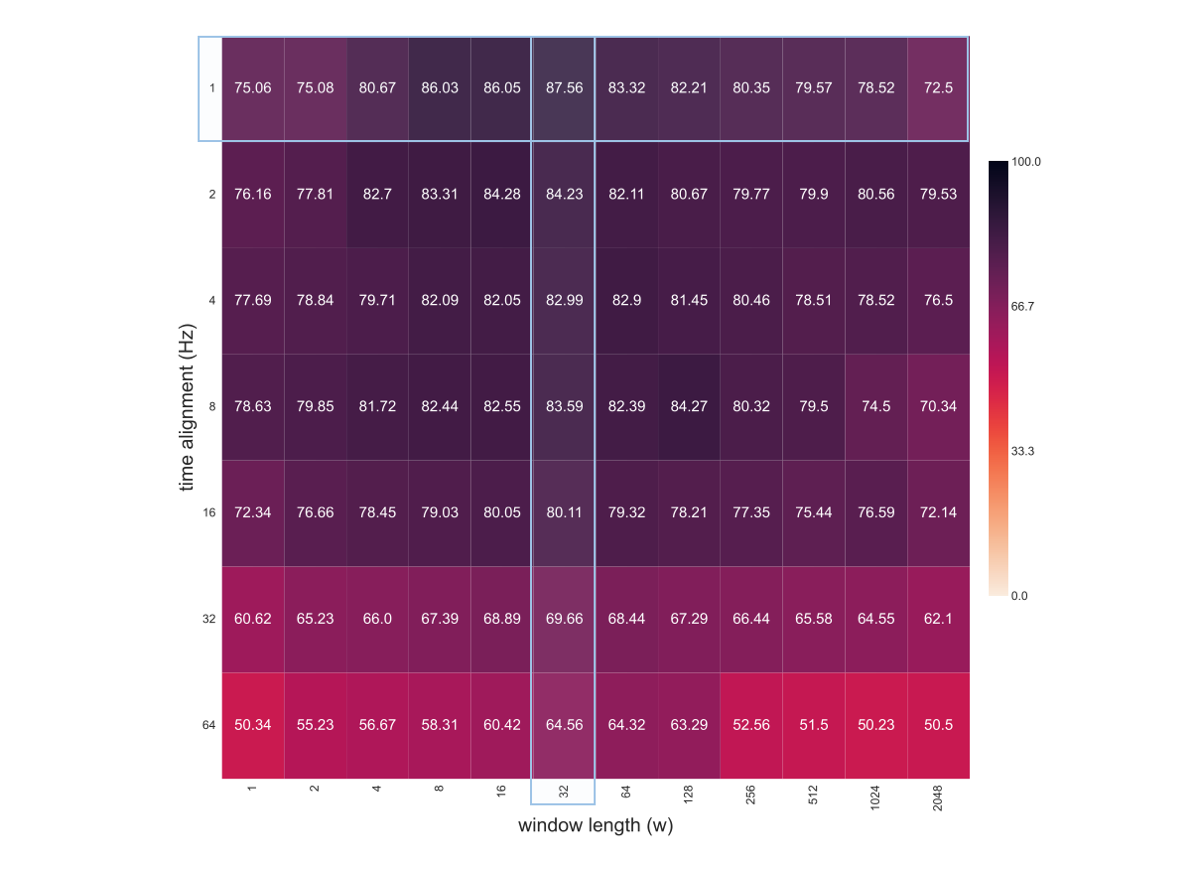

Supplement: Multimedia Appendix 2 [file mhealth_v11i1e45405_app2.png]

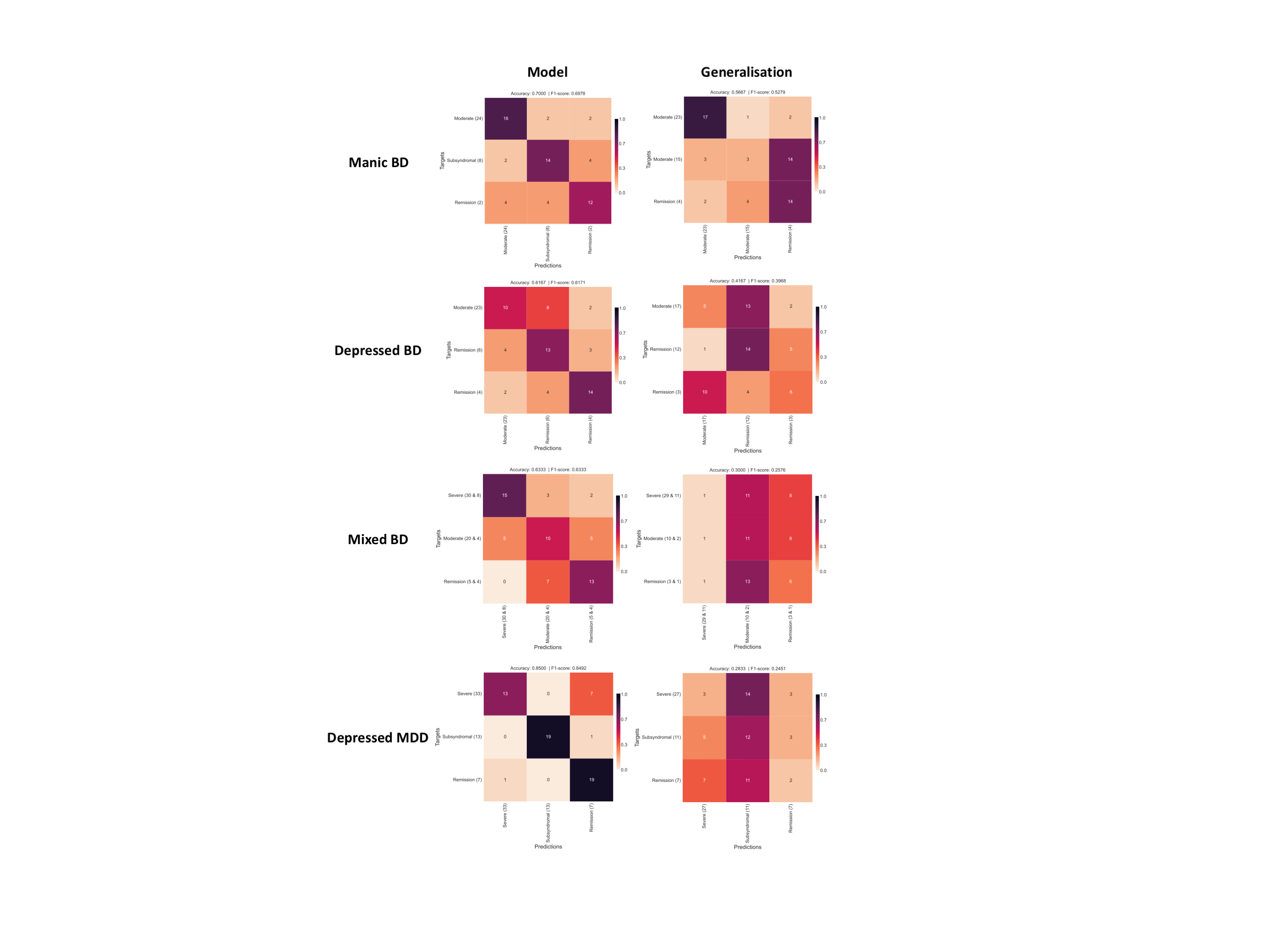

Supplement: Multimedia Appendix 3 [file mhealth_v11i1e45405_app3.png]

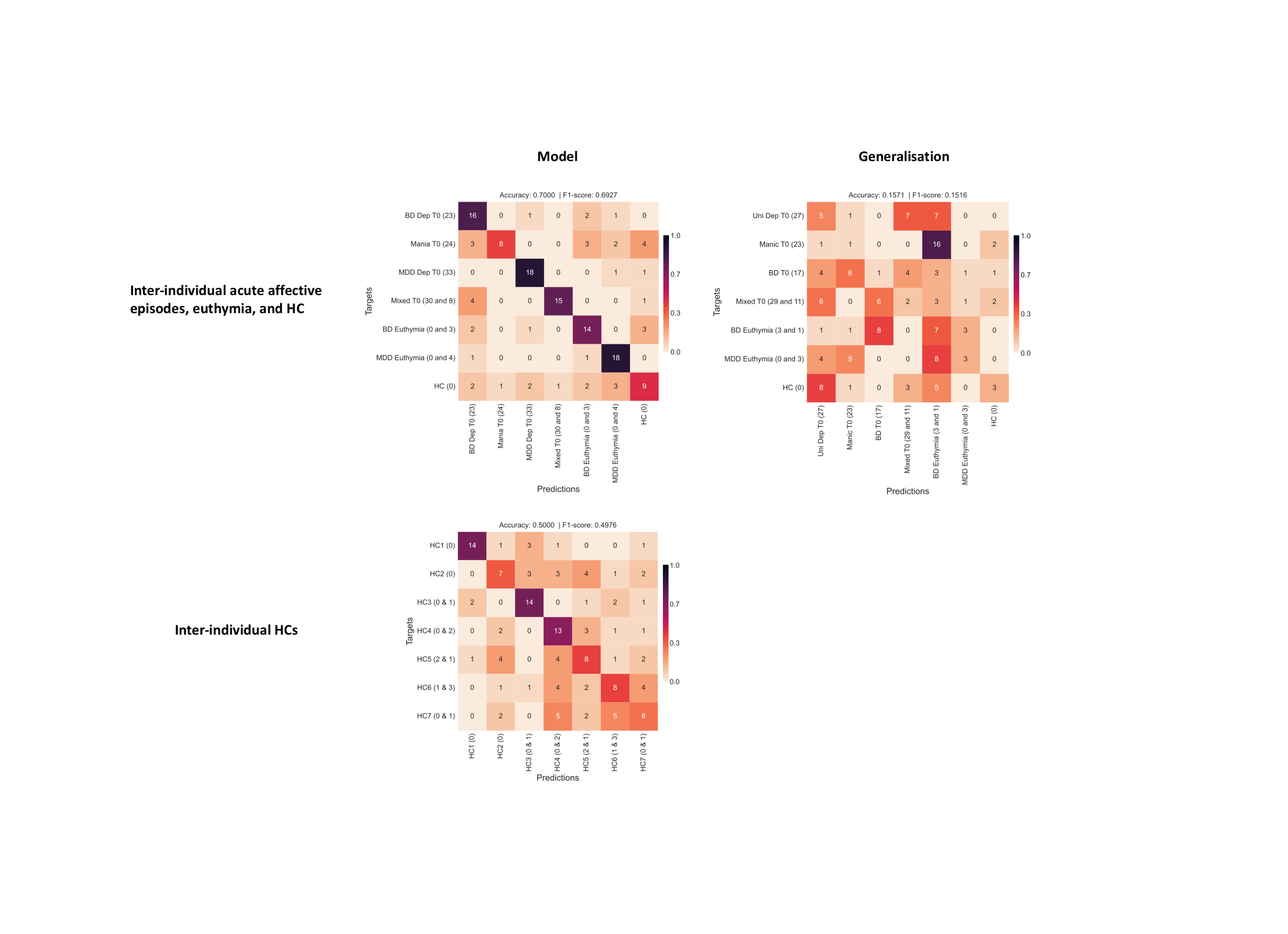

Supplement: Multimedia Appendix 4 [file mhealth_v11i1e45405_app4.png]
